# Severe anaphylaxis after pelvic examination: a case report of dual latex and chlorhexidine allergies

**DOI:** 10.1186/s13223-019-0335-4

**Published:** 2019-03-29

**Authors:** Mongkhon Sompornrattanaphan, Piyawut Kreetapirom, Yuttana Srinoulprasert, Duangjit Kanistanon, Anchalika Klinniyom, Chamard Wongsa, Torpong Thongngarm

**Affiliations:** 1 Division of Allergy and Clinical Immunology, Department of Medicine, Faculty of Medicine Siriraj Hospital, Mahidol University, Bangkok, Thailand; 2 Division of Pediatrics, Chaophya Hospital, Bangkok, Thailand; 3 Department of Immunology, Faculty of Medicine Siriraj Hospital, Mahidol University, Bangkok, Thailand; 4 Department of Pharmacy, Siriraj Hospital, Mahidol University, Bangkok, Thailand

**Keywords:** Pelvic examination, Anaphylaxis, Latex allergy, Chlorhexidine, Hypersensitivity

## Abstract

**Background:**

Natural rubber latex and chlorhexidine have previously been identified as causative substances in perioperative anaphylaxis. A pelvic examinations is generally considered noninvasive, however, this procedure is rarely associated with severe allergic reactions. We reported a rare case of dual latex and chlorhexidine allergies which caused anaphylaxis after pelvic examination in a woman with a history of latex-related fruits allergy.

**Case presentation:**

A 54-year-old woman had severe anaphylaxis after a pelvic examination due to dual latex and chlorhexidine (CHX) allergies. The gynecologist used CHX for the vaginal preparation and wore latex-containing gloves with lubricating gel during the examination. In vivo and in vitro tests revealed CHX sensitization by a positive skin prick test to chlorhexidine at a very low concentration (0.002 mg/mL), and a positive basophil activation test to CHX. Latex allergy was confirmed by a positive specific IgE to latex and a positive glove-use test at 20 min. An analysis of specific IgE to latex component revealed positive results for Hev b 1, 5, 6.02, and 11. As she also had a past history of fruit allergy, prick-to-prick testing with latex-related fruits was performed. The results were positive for avocado, banana, jackfruit, kiwi, and longan.

**Conclusions:**

Concomitant mucosal exposure of both natural rubber latex and CHX in highly sensitized patients during pelvic examinations can lead to severe anaphylaxis. Pre-procedural screening for an allergy to latex or CHX, or to any other allergen, should be performed in patients where there is suspicion of a specific allergy due to a previous allergic reaction. Increased awareness of these two allergens in all healthcare settings may improve patient safety.

## Background

The incidence of perioperative anaphylaxis is being increasingly reported in the literature. Natural rubber latex (NRL) and chlorhexidine (CHX) have previously been identified as causative substances. These exposures have usually been associated with anaphylaxis during an operation and, less frequently, during a noninvasive procedure [1, 2]. A pelvic examination of a female is generally considered noninvasive; this procedure, however, is rarely associated with severe allergic reactions [3].

## Case presentation

We report on a case of severe anaphylaxis after a pelvic examination performed on a 54-year-old Thai woman. She had had a spinal cord tumor surgically removed 4 years prior to the episode of anaphylaxis. She consequently had spastic paraplegia and became bed-bound. She developed one chronic pressure sore at the labia majora, for which she underwent multiple debridements. NRL gloves and CHX were regularly used in the debridements. Three days prior to the episode of anaphylaxis, she complained of vaginal spotting. A pelvic examination was done to diagnose the pelvic pathology. The gynecologist used CHX for the vaginal preparation and wore NRL gloves with lubricating gel during the examination. No other medications were administered during peri-procedural period. Approximately 5 min after the examination, she began to complain of “feeling unwell” with mild vaginal pruritus. She rapidly developed generalized hives and flares, swollen eyelids, and hypotensive syncope, all within 30 min. She was diagnosed with severe anaphylaxis. Intravenous fluid and intramuscular epinephrine were promptly administered. All symptoms improved without a biphasic reaction.

Additional history revealed that she had a history of minimal pruritus after direct skin contact with latex gloves during a bed bath procedure 1 month before. Although NRL gloves and CHX as a disinfectant were regularly used in her debridements, she had never had a history of a systemic allergic reaction after a debridement. Since the age of 50, she had had a history of fruit allergy (including bananas, longans, and jackfruit) associated with symptoms of pruritus of the lips, urticarial rashes, and angioedema. She had never eaten avocado, kiwi, or other latex-related fruits.

Investigations into the cause of the anaphylaxis were done thoroughly 6 weeks after the episode. The results are summarized in Table 1.

**Table 1:** Investigations performed in this patient with anaphylaxis after pelvic examination

Skin prick test^a^	Prick-to-prick test^b^	Specific IgE^c^
Chlorhexidine digluconate (0.002 mg/mL): 7 × 7 mm with flare	Longan: 9 × 5 mm with flare Jackfruit: 7 × 7 mm with flare	Latex: 45.90 kAU/L rHev b 1: 1.79 kAU/L
K-Y jelly: negative	Cultivated banana: 4 × 3 mm with flare	rHev b 3: 0.32 kAU/L
Positive control: 8 × 8 mm with flare	Cavendish banana: 7 × 6 mm with flare	rHev b 5: 41.5 kAU/L
Negative control: negative	Avocado: 6 × 5 mm with flare Kiwi: 6 × 5 mm with flare Chestnut: negative	rHev b 6.02: 32.1 kAU/L rHev b 8: 0 kAU/L rHev b 11: 2.96 kAU/L

*IgE* immunoglobulin E, *rHev b* recombinant Hevea brasiliensis allergen, *kAU/L* kilo allergy unit per liter, *K-Y jelly* water-based, water-soluble personal lubricant, Johnson & Johnson

^a^Normal saline and histamine (10 mg/mL) were used as negative and positive controls, respectively. We did not perform a latex skin test due to unavailability of a standard solution

^b^Prick-to-prick test by using fresh fruit

^c^Solid-phase immunoassay: ImmunoCAP

**Fig. 1:**
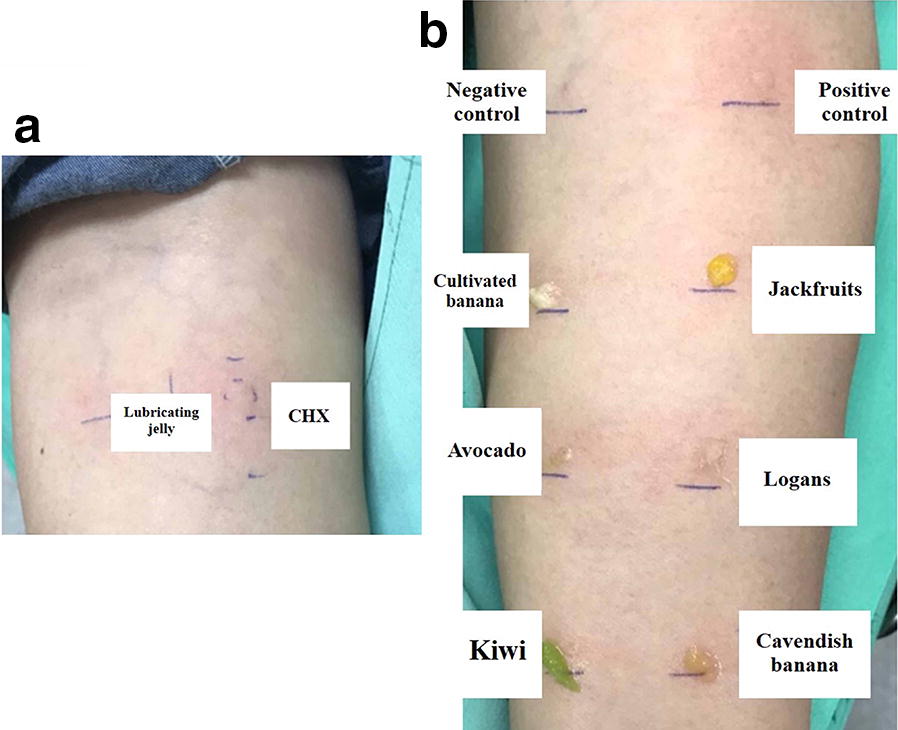
Skin test results. **a** Skin prick test to lubricating jelly and chlorhexidine. *CHX* chlorhexidine. **b** Prick-to-prick test to latex-related fruits. Normal saline and histamine (10 mg/mL) were used as negative and positive controls, respectively

Given the patient’s history of severe index reaction, we initially performed skin prick testing with CHX at a concentration of 0.002 mg/mL, which proved to be positive. A basophil activation test showed an increased expression of CD203c in basophils when stimulated with CHX. Its stimulation indices were 4.03 and 4.52 when whole blood was stimulated with CHX at the concentrations of 0.1 and 0.3 mcg/mL, respectively. Specific IgE to latex, using ImmunoCAP, was positive at 45.90 kAU/L. We analyzed specific IgE to latex component, which showed positive results for Hev b 1, 5, 6.02, and 11 (Phadia AB, Uppsala, Sweden). Prick-to-prick testing with latex-related fruits was positive for avocado, banana, jackfruit, kiwi, and longan (Fig. 1). A glove-use test revealed contact erythema with concurrent pruritus on the fingertip at 20 min.

## Discussion and conclusions

This is a case of severe anaphylaxis due to dual NRL and CHX allergies. Latex-fruit syndrome, an association of between latex allergy and plant-derived foods allergy, was also diagnosed. The cross-reactivity was possibly due to IgE reactivity to structurally similar epitopes on different proteins, which was confirmed by the component-resolved diagnosis. These peptides/proteins from NRL and fruits are structurally related, including Hev b 5, 6, 7, and 11. Physicians should thus be aware of the association between latex and food allergens [4]. Ten percent of fruit-allergic patients are at risk because they carry a probability of an associated latex allergy [1].

Reports of adverse reactions to CHX have increased over recent decades [5]. Previous studies have reported that patients presenting with CHX anaphylaxis had histories of previous mild urticarial reactions to CHX. It is recognized as a “hidden” culprit, causing anaphylaxis in the perioperative period, and it has occasionally been reported as being associated with a pelvic examination [2, 3]. Either topical exposure or invasive administration of CHX could lead to allergic reactions in sensitized individuals [5, 6]. Our patient underwent multiple surgical interventions and repeated exposures to CHX during wound debridement and urinary catheterization. We consider these as risk factors for the development of a CHX allergy. Caution in the routine use of CHX to mucous membranes has previously been suggested due to the potential risk of anaphylactic reactions [7].

This case report has the potential to offer an early signal at the point of care that informs the current practice. Firstly, the potential for anaphylaxis on vaginal contact with NRL and CHX is well-established. Current practice guidelines, however, focus on the identification of high-risk patients only in an operative setting [8]. Most of the recommendations do not mention allergic screening before a pelvic examination. Pre-procedural allergy screening should thus be expanded to noninvasive procedures that involve mucosal manipulation. Secondly, there is a range of etiologies of hypotension in obstetric and gynecologic patients, and recognition of anaphylaxis and initiation of anaphylaxis-specific treatment is often delayed compared with non-obstetric and gynecologic cases [9]. Epinephrine and other resuscitative measures should, therefore, be available in the setting in which such procedures are performed. Finally, we have reported the first case of dual NRL and CHX allergies with concomitant latex-fruit syndrome. There have been cases with a CHX allergy which may also exhibit positive results for other allergen testing. We thus suggest that if CHX is positive on testing, the other relevant exposures should be tested [9].

In conclusion, concomitant mucosal exposure of both NRL and CHX in highly sensitized patients during pelvic examination can lead to severe anaphylaxis. Pre-procedural screening for an allergy to latex or CHX, or to any other allergen, especially fruits, should be performed in patients where there is suspicion of a specific allergy due to a previous allergic reaction. Increased awareness of these two allergens in all healthcare settings may improve patient safety.

## Patient’s perspective section

I came to the hospital for a pelvic examination appointment. I had not expected to have an allergic event. The experience of allergic reaction was frightful. The feeling of being unwell was the first symptom that I tried to explain to the physician. Then I lost consciousness and found myself in the intensive care unit after recovery. After the complete investigation, I was diagnosed with the latex-fruit syndrome and chlorhexidine allergy, both of which were unfamiliar to me. The physician told me that having latex and fruits allergy is something that has previously reported in medical journals.

I wish that the reaction could have been prevented if a history of fruits allergy had been considered significant in the initial evaluation before starting the procedure. I hope that my case will cause everyone to focus on the history of fruits allergy before using the latex-containing products.

## Abbreviations

NRL: natural rubber latex
CHX: chlorhexidine
IgE: immunoglobulin E
kAU/L: kilo allergy unit per liter
